# Modulating the Mechanical Activation of TRPV4 at the Cell-Substrate Interface

**DOI:** 10.3389/fbioe.2020.608951

**Published:** 2021-01-18

**Authors:** Setareh Sianati, Lioba Schroeter, Jessica Richardson, Andy Tay, Shireen R. Lamandé, Kate Poole

**Affiliations:** ^1^EMBL Australia Node in Single Molecule Science and Cellular and Systems Physiology, Faculty of Medicine, School of Medical Sciences, University of New South Wales, Sydney, NSW, Australia; ^2^Murdoch Children's Research Institute and Department of Paediatrics, University of Melbourne, Parkville, VIC, Australia

**Keywords:** TRPV4, mechanotransduction, mechanically activated ion channel, cell-substrate interface, mechanics

## Abstract

Ion channels activated by mechanical inputs are important force sensing molecules in a wide array of mammalian cells and tissues. The transient receptor potential channel, TRPV4, is a polymodal, nonselective cation channel that can be activated by mechanical inputs but only if stimuli are applied directly at the interface between cells and their substrate, making this molecule a context-dependent force sensor. However, it remains unclear how TRPV4 is activated by mechanical inputs at the cell-substrate interface, which cell intrinsic and cell extrinsic parameters might modulate the mechanical activation of the channel and how mechanical activation differs from TRPV4 gating in response to other stimuli. Here we investigated the impact of substrate mechanics and cytoskeletal components on mechanically evoked TRPV4 currents and addressed how point mutations associated with TRPV4 phosphorylation and arthropathy influence mechanical activation of the channel. Our findings reveal distinct regulatory modulation of TRPV4 from the mechanically activated ion channel PIEZO1, suggesting the mechanosensitivity of these two channels is tuned in response to different parameters. Moreover, our data demonstrate that the effect of point mutations in TRPV4 on channel activation are profoundly dependent on the gating stimulus.

## Introduction

Ion channels that are directly activated by mechanical inputs are emerging as important cellular force sensors in a wide variety of cells and tissues (Wu et al., 2017). When mechanical perturbations are applied to cells at the cell-substrate interface, ionic currents can be evoked that depend on a number of different ion channels, including TRPV4, as well as PIEZO1, PIEZO2, and TACAN (Poole et al., 2014; Servin-Vences et al., 2017; Wetzel et al., 2017; Bavi et al., 2019; Sianati et al., 2019; Beaulieu-Laroche et al., 2020). TRPV4 is a polymodal, Ca^2+^-permeable, nonselective cation channel that is broadly expressed throughout the body (Nilius and Voets, 2013; White et al., 2016). TRPV4 has a tetrameric structure; each subunit consisting of six transmembrane (TM) domains, a pore loop between TM5 and TM6 and cytoplasmic N- and C-terminal tails. The N terminus contains six ankyrin repeat domains and the C terminus has a binding site for actin and tubulin (Goswami et al., 2010; Deng et al., 2018). The activation of TRPV4 has been implicated in various biological processes such as osmosensation (Liedtke and Friedman, 2003; Suzuki et al., 2003b; Liedtke, 2005; Lechner et al., 2011), bone homeostasis (O'Conor et al., 2014), arterial dilation (Mendoza et al., 2010), bladder voiding (Gevaert et al., 2007) and nociception (Alessandri-Haber et al., 2003, 2004). The precise mode of activation of TRPV4 in these different biological settings is largely unknown due to the fact that TRPV4 is polymodal: that is, TRPV4 is activated by diverse stimuli including osmotically-induced cell swelling (Strotmann et al., 2000; Vriens et al., 2004), shear stress (Köhler et al., 2006), moderate heat (Güler et al., 2002; Watanabe et al., 2002b), low pH (Suzuki et al., 2003b) and various chemical ligands (Watanabe et al., 2002a, 2003). The activation of TRPV4 by different stimuli suggests that this ion channel exhibits distinct gating pathways. For example, activation of TRPV4 by osmotic cell swelling requires phospholipase A2 (PLA2) activity, whereas agonist 4α-PDD and heat activate TRPV4 in a PLA2-independent manner but require a tyrosine in the third transmembrane domain of TRPV4 (Vriens et al., 2004). A single point mutation at Y621 in TM5 exhibits opposing effects on channel activation in response to different stimuli, resulting in decreased channel sensitivity in response to 4α-PDD and heat but increased activation in response to cell swelling (Klausen et al., 2014). Moreover, interaction of PACSIN 3 with the N-terminal domain of TRPV4 inhibits channel activation by cell swelling and heat but has no effect on Ca^2+^ influx induced by 4α-PDD (D'hoedt et al., 2008).

The potential importance of TRPV4 activation in *in vivo* mechanical sensing is highlighted by the influence of TRPV4 on homeostatic maintenance of both cartilage and bone, two tissues that are regulated by mechanical inputs. Mutations in TRPV4 have been linked to different pathophysiological conditions such as skeletal dysplasia, arthropathy and peripheral neuropathy (Lamandé et al., 2011). Moreover, global deletion of TRPV4 in mice increases bone mass due to impaired osteoclast differentiation and accelerates the progression of age-dependent and obesity-induced osteoarthritis (Clark et al., 2010; O'Conor et al., 2013). Conversely, loss of TRPV4 in chondrocytes in adulthood protects mice from aging-associated osteoarthritis (O'Conor et al., 2016). Such observations, in addition to the expression pattern of TRPV4 in many mechanosensitive tissues, indicate the involvement of TRPV4 in mechanotransduction.

However, it has been challenging to demonstrate that TRPV4 acts as a primary transducer of mechanical inputs. The *in vitro* study of mechanical activation of ion channels in the cell membrane requires experimental approaches to simultaneously monitor ionic flux whilst applying an appropriate mechanical input to the cell. Many studies utilize high-speed pressure clamp to locally stretch the plasma membrane or a blunt probe to cause cellular indentation (Coste et al., 2010; Lee et al., 2014; Nourse and Pathak, 2017; Wu et al., 2017). To complement these techniques, we have developed a methodology to apply stimuli directly to the cell-substrate interface. Cells are cultured on elastomeric pillar arrays and individual elements subjacent to the cell can be deflected, whilst the cellular response is monitored using whole-cell patch-clamp (Poole et al., 2014; Sianati et al., 2019). *In vitro* studies of TRPV4 mechanical activation using these three techniques showed that TRPV4 poorly responds to membrane stretch applied by high-speed pressure clamp (Loukin et al., 2010; Nikolaev et al., 2019) and completely fails to respond to cellular indentation (Servin-Vences et al., 2017). However, when pillar arrays are used to apply fine deflections to connections between a cell and its substrate (or cell-substrate interface), TRPV4-mediated currents are activated (Servin-Vences et al., 2017; Tay et al., 2018; Sianati et al., 2019). Nano-scale deflections of regions of the cell-substrate interface activate TRPV4 in both primary chondrocytes and when TRPV4 is expressed heterologously in HEK-293T cells. These deflection-evoked currents exhibit latencies below 2 ms, and do not depend on PLA2, suggesting that TRPV4 is directly activated by the mechanical input (Servin-Vences et al., 2017). In addition, mechanically activated (MA) currents evoked by substrate deflection are not outwardly rectifying, unlike 4α-PDD, heat and cell swelling activated currents (Vriens et al., 2004; Servin-Vences et al., 2017).

That TRPV4 can be efficiently activated by cell-substrate deflection but not cellular indentation or membrane stretch highlights the fact that TRPV4 responds to mechanical stimuli in specific cellular and physical contexts. In order to better understand activation of TRPV4 in the cell-substrate interface, we investigate the impact of the cytoskeleton and substrate mechanics on TRPV4 mechanotransduction. Furthermore, we measured deflection-activated currents of TRPV4 mutant channels that are associated with either gain- or loss-of-function in response to chemical agonists.

## Materials and Methods

### Cell Culture

HEK-293T Flp-In T-REx cells stably expressing TRPV4 were cultured in Dulbecco's Modified Eagle Medium (DMEM, Gibco) supplemented with 10% fetal bovine serum (Gibco), 1% Penicillin/Streptomycin and selected with 15 μg/ml blasticidin and 100 μg/ml hygromycin. Cells were maintained in a humidified incubator at 37°C, 5% CO_2_. Expression of TRPV4 wild-type and mutant channels was induced by application of tetracycline (1 μg/ml, 4 h), as previously described (Lamandé et al., 2011).

### Pillar Array Fabrication

Pillar array casting and preparation were conducted as previously described (Poole et al., 2014; Sianati et al., 2019). Briefly, positive silicon masters (obtained from Bonda Technology Pte Ltd., Singapore) were silanized using vapor phase Trichloro(1H,1H,2H,2H-perfluorooctyl)silane (Sigma-Aldrich) to create a hydrophobic surface. Negative masters were then cast from the positive masters using polydimethylsiloxane (PDMS) (Sylgard 184, Dow Corning) at a ratio of 1:10, and cured for 15 min at 110°C. To cast pillar array substrates, the silanized negative masters were coated with degassed PDMS (at ratio of 1:10 or 1:5 for stiffer substrates) and incubated for 30 min. Each coated master was covered with an oxygen plasma-activated glass coverslip. Pillar arrays were cured for 1 h (or 2 h for stiffer substrate) at 110°C and then peeled away from the masters. Before culturing the cells, pillar arrays (attached to coverslip) were activated with oxygen plasma for 90 s (Zepto plasma system, Diener, Germany). To achieve robust adhesion, cells were seeded on the pillar arrays 24 h prior to experiments.

Masters for casting arrays with distinct mechanical properties have been previously described (Bavi et al., 2019; Sianati et al., 2019). In brief, substrate roughness was manipulated by altering the geometry of the array, according to the equation:

(1) $$\begin{array}{l} \varepsilon =1+\frac{\pi dH}{S^{2}} \end{array}$$

Where *d* = diameter, *H* = height and *S* the center-to-center inter-pillar spacing.

Substrate stiffness was controlled by changing the spring constant of the individual elements of the array by changing either the elasticity *E* of the material or the geometrical dimensions of the pilus, in accordance with the equation:

(2) $$\begin{array}{l} k=\frac{3\pi Ed^{4}}{64H^{3}}. \end{array}$$

### Patch-Clamp Recordings on Pillar Arrays

Whole-cell patch-clamp data were acquired from cells seeded on pillar arrays using Axopatch 200B amplifier with pClamp 10 software at holding potential of −60 mV. Data were sampled at 5 kHz using Axon Digidata 1550B digitizer (Molecular devices, USA). Recording pipettes (3–5 MΩ) were filled with intracellular solution containing (in mM) 135 KCl, 10 NaCl, 1 MgCl_2_, 1 EGTA, and 10 HEPES; pH 7.3, 280–285 mOsm. Extracellular solution contained (in mM) 140 NaCl, 4 KCl, 2 CaCl_2_, 1 MgCl_2_, 4 glucose and 10 HEPES; pH 7.4, 290–295 mOsm.

Series resistance was monitored throughout the experiments and remained < 15 MΩ. Data were analyzed using Clampfit software (Molecular Devices, USA). Inward current amplitude was measured as the difference between the baseline and the peak of the current. The latency was quantified as the time delay between the stimulus application and onset of the current. The activation time constant (τ_1_) and inactivation time constant (τ_2_) were calculated by a mono-exponential fit of the current rise and current decay, respectively. Cytochalasin D (2.5 μM) and Nocodazole (10 μM) were diluted in the extracellular solution 15 and 30 min prior to experiments, respectively. Treated cells were discarded 30 min after the first recording.

To generate mechanical stimuli at cell-substrate contact points, a series of deflections ranging from 1 to 1,000 nm were applied to an individual pilus subjacent to the cell. The stimulator was prepared by heat-polishing a glass micropipette (to seal the tip) and was driven by a MM3A-LS micromanipulator (Kleindiek Nanotechnik, Germany). To calculate the stimulus size, bright field images were acquired before and during stimulation using a 40x/0.6 NA objective on Nikon *ti*-e inverted microscope. The center of the pilus (prior and during stimulus) was determined using a 2D-gaussian fit of intensity values (Igor, Wavemetrics, USA) and the magnitude of pilus deflection was analyzed by comparing the displacement of the center of the pilus between two successive images.

### Calcium Imaging

HEK293T cells expressing WT or the mutant TRPV4 variants were cultured on PLL-coated glass coverslips and incubated with 1 μM Cal_520_ (AAT Bioquest, USA), a calcium sensitive dye, for 1 h. Cells were perfused with 1 μM GSK1016790A for 1 min and then were washed out with extracellular solution for 10 min. Calcium images were captured every 10 s using Nikon *ti-*e inverted microscope and were analyzed using ImageJ. Calcium signal was calculated according to the equation ΔF/F = (F–F_0_)/F_0_ where F is mean intensity of fluorescence and F_0_ is the baseline fluorescence prior to application of the agonist.

### Immunoblotting and Cell-Surface Biotinylation

To detect TRPV4 variants at the plasma membrane, the cell surface fraction was biotinylated and subsequently isolated following established protocols (Lamandé et al., 2011; Tarradas et al., 2013). Briefly, stably transfected Flp-in TREx HEK-293T cells attached to PLL-coated 10 cm culture dishes were treated with 0.1 μg/ml tetracycline for 16 h to induce expression of TRPV4 variants. The cell surface fraction was labeled, on ice, with freshly prepared 2.5 mg/ml EZ-link Sulfo-NHS-LC-LC-biotin (21338, ThermoFisher Scientific) in DPBS containing Ca^2+^. After quenching with 100 mM glycine, cells were lyzed using RIPA buffer containing protease inhibitor cocktail. A portion of this lyzed sample was reserved as the “input” sample. The biotinylated fraction was then isolated using NeutrAvidin Ultralink Resin (53150, ThermoFisher Scientific). After recovery from the NeutrAvidin beads, samples were prepared as for gel electrophoresis by mixing with Bolt LDS sample buffer and Bolt reducing agent (B0007 and B0009, respectively, ThermoFisher Scientific). Samples were separated on a 10% Bolt Bis-Tris Plus gel (NW00102, ThermoFisher Scientific), transferred to a PVDF membrane and subjected to standard antibody detection. TRPV4 variants were detected using rabbit polyclonal anti-TRPV4 (ab191580, Abcam, 1 ug/ml) for 1 h at room temperature and HRP-linked anti-rabbit IgG (7074, Cell Signaling Technologies, 1:1,000). To control for protein loaded, membranes were re-probed using anti-alpha-Tubulin (9099, Cell Signaling, Technologies, 1:1,000) over night at 4°C, after membrane stripping.

### Imaging

To visualize TRPV4 localization, cells were transiently transfected with a plasmid encoding TRPV4-YFP, using Fugene HD (E2311, Promega) per manufacturer's instructions. Cells were then washed with PBS and fixed for 10 min with 4% paraformaldehyde (PFA). Cells were permeabilized with 0.01% TritonX-100 and labeled with DyLight 594 Phalloidin, as per manufacturers instructions (12877, Cell Signaling Technology). Cells were imaged using either a Zeiss Axioskop or Leica SP8 with a 40x objective. Resulting images were analyzed using ImageJ analysis software (Schneider et al., 2012). In order to assess the localization of TRPV4 and actin to pillar structures, each pilus was scored according to whether the protein formed a ring around the total circumference of the pilus, a partial circumference or if there was no increase in signal around the pilus. The TRPV4-YFP and phalloidin-594 signals were then compared and scored as a colocalization when similar structures were observed around an individual pilus.

### Statistical Analysis

The current amplitudes generated by substrate deflection were binned by stimulus size into the following groups: 0–10, 10–50, 50–100, 100–250, 250–500, and 500–1,000 nm, as previously described (Poole et al., 2014; Sianati et al., 2019). The response data were averaged within each bin (for each stimulation point) and then were averaged across the cells. Data was collected from samples prepared on at least three separate days. Wherever appropriate, data were analyzed using one-way or two-way ANOVA, followed by Tukey's or Sidak's *post hoc* tests or unpaired two-tailed *t*-test with significance set at *p* < 0.05. The results for each experiment are presented as the mean ± s.e.m. The data were analyzed using GraphPad Prism 7 software (GraphPad Software, Inc., San Diego, CA).

## Results

### Substrate Roughness Does Not Mediate TRPV4 Deflection-Activated Currents

**Figure 1:**
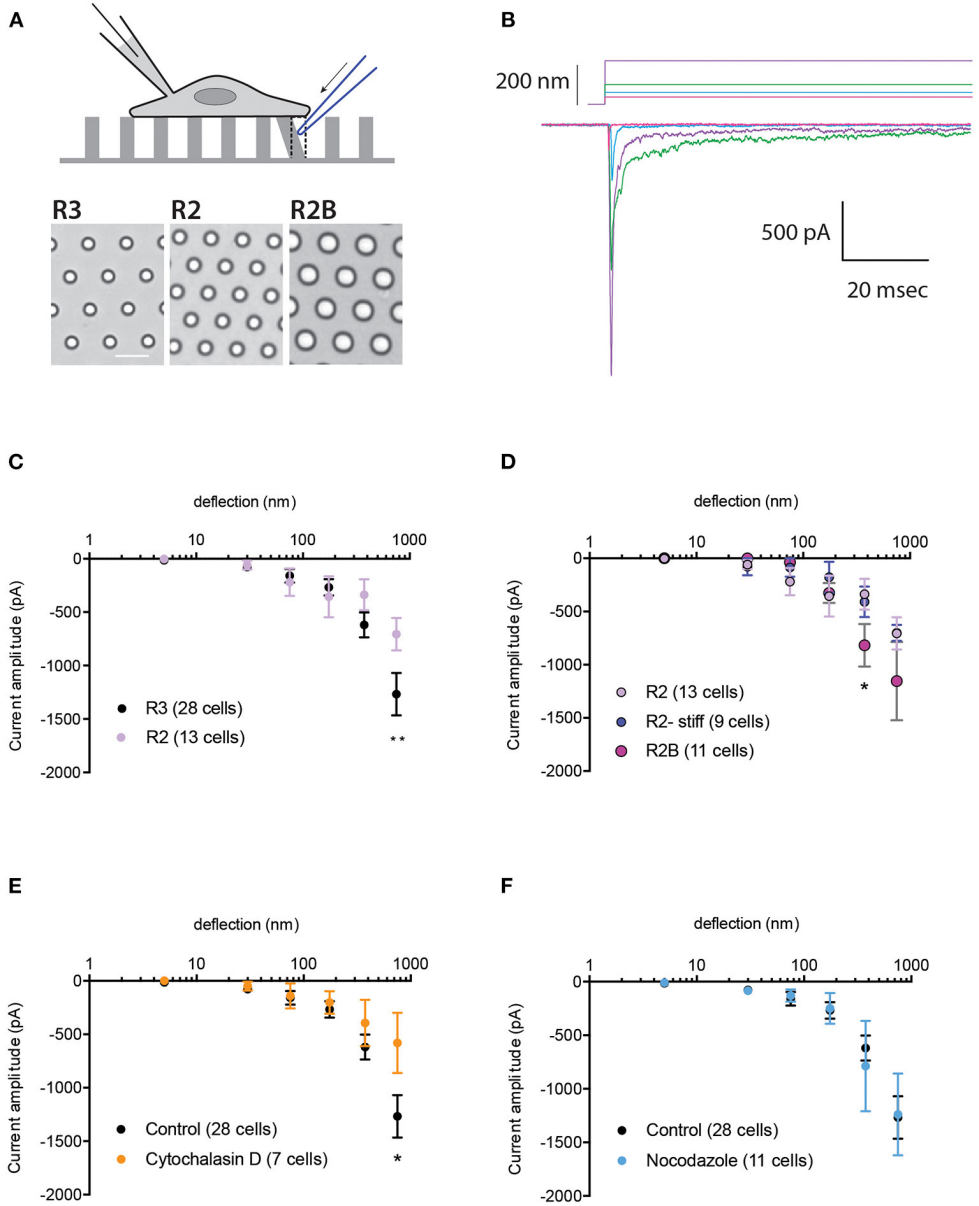
Impact of substrate mechanics and cytoskeleton on TRPV4-mediated currents. **(A)** Schematic representation of pillar array experiment. Cells are cultured on deformable arrays and stimuli are applied at cell-substrate interface by deflecting a pilus subjacent to the cell using a glass probe (in blue). The resulting channel activity is concurrently monitored using whole-cell patch-clamp. Bottom panels show bright-field images of the substrates R3, R2 and R2B, scale bar 10μM. **(B)** Representative traces of TRPV4-WT mediated currents in response to increasing deflections from 28 to 212 nm on the R3 substrate. **(C)** Stimulus-response plot for TRPV4-WT cells cultured on R3 and R2 substrates (ordinary two-way ANOVA, **p = 0.009) **(D)** Stimulus-response plot for TRPV4-WT cells cultured on R2, R2 stiff and R2B (ordinary two-way ANOVA, *p = 0.019). Stimulus-response plots for TRPV4-WT cultured on R3 substrate after treatment with **(E)** 2.5 μM cytochalasin D and **(F)** 10 μM nocodazole compared to the control (ordinary two-way ANOVA, *p = 0.01). Data are presented in mean ± s.e.m, number in brackets indicates number of cells.

Changes in substrate mechanics can modulate the activation of the *bone fide* mechanically activated (MA) channel, PIEZO1 (Bavi et al., 2019). To investigate whether the mechanical activation of TRPV4 is also regulated by the mechanics of the substrate, HEK-293T cells expressing TRPV4 were cultured on elastomeric pillar arrays with different roughness or stiffness, as previously described (Bavi et al., 2019) (Figure 1A). In order to generate comparable protein expression levels across various cell lines, we utilized HEK-293T Flp-In T-REx cells. In these cells, only one copy of the transgene (encoding TRPV4 variants) is integrated into each line. Expression of TRPV4 variants was induced using tetracycline in order to generate samples with consistent expression levels and to prevent calcium toxicity that can result from continuous overexpression of TRPV4. We first tested whether changes in substrate roughness or stiffness impacted TRPV4-mediated currents evoked by substrate deflection. To manipulate substrate roughness (ε), pillar arrays with differences in the center-to-center pillar spacing, were utilized (R3, with ε: 1.53 vs. rougher substrate R2, with ε: 1.80) (Bavi et al., 2019). The channel activity in cells seeded on the arrays was recorded using whole-cell patch-clamp, whilst the mechanical stimuli were applied at the cell-substrate interface by deflecting an individual pilus subjacent to the cell (Figures 1A,B). The current amplitude data from each cell were binned according to the stimulus size and averaged within each bin and then across cells. Stimulus-response curves were generated by plotting the average of current amplitudes against the corresponding pillar deflection range. There was no significant difference in TRPV4-mediated currents at molecular-scale deflections (1–375 nm) between the two substrates (Figure 1C). However, there was a moderate increase in current amplitude from cells cultured on R3 compared to R2 at larger deflections (up to 1,000 nm). Various kinetic parameters of the mechanically evoked currents were analyzed; latency between stimulus and the onset of the current, the activation time constant and the inactivation time constant (current decay). There were no differences in any of these kinetic parameters on the different substrates (Supplementary Table 1).

To assess the impact of substrate stiffness on TRPV4-mediated currents, we used two different approaches. First, the stiffness of the R2 pillar arrays was increased by doubling the ratio of curing agent to elastomer. Analysis of current amplitudes showed no difference in TRPV4 currents in response to deflections ranging from 1 to 1,000 nm between these two substrates (Figure 1D). Changes in the pillar array stiffness had no effect on current kinetics, similar to those quantified from substrates with different roughness. We next increased the substrate stiffness by utilizing the R2B substrate that has a larger pillar diameter but similar roughness to R2, leading to consistent substrate roughness and bulk substrate elasticity but an increase in the spring constant (*k*) of the individual pillar elements within the array (R2: *k* = 0.89 nN/nm, R2B: 4.27 nN/nm) (Bavi et al., 2019). Changes in substrate stiffness did not affect TRPV4-mediated currents in response to deflections of 1–250 nm. However, there was an increase in current amplitude on R2B vs. R2 in response to larger stimuli that was significant within the range of 250–500 nm (Figure 1D, ordinary two-way ANOVA, Sidak's multiple comparison tests, *p* = 0.019). It should be noted that increasing the pillar diameter may also result in greater channel numbers around the circumference of each individual pilus which could account for the moderate increase in the current amplitude. As such, we conclude that mechanical properties (roughness and stiffness) of the substrate only exhibit minor effects on the mechanical activation of TRPV4 when large stimuli are applied at the cell-substrate contact point, unlike PIEZO1 where we observed an increase in channel sensitivity to molecular-scale substrate deflections on the substrate with lowest roughness, R3 (Bavi et al., 2019). These data suggest that MA ion channels have diverse abilities to sense and respond to their mechanical microenvironment.

### TRPV4-Mediated Currents Are Not Dependent on Cytoskeletal Structure

Our recent work revealed that the actin cytoskeleton modulates mechanically evoked PIEZO1-dependent currents in response to externally applied deflections of the cell-substrate interface (Bavi et al., 2019). In addition, PIEZO1-mediated currents that are dependent on cell generated forces at cell-substrate connections also require an intact actin cytoskeleton (Pathak et al., 2014; Ellefsen et al., 2019). TRPV4 has been demonstrated to interact directly with both actin and microtubules via the C-terminus of the channel (Suzuki et al., 2003a; Becker et al., 2009; Goswami et al., 2010; Huai et al., 2012; Lyons et al., 2017). Given these data, we investigated whether the TRPV4-mediated currents are also dependent on intact cytoskeletal structures.

**Figure 2:**
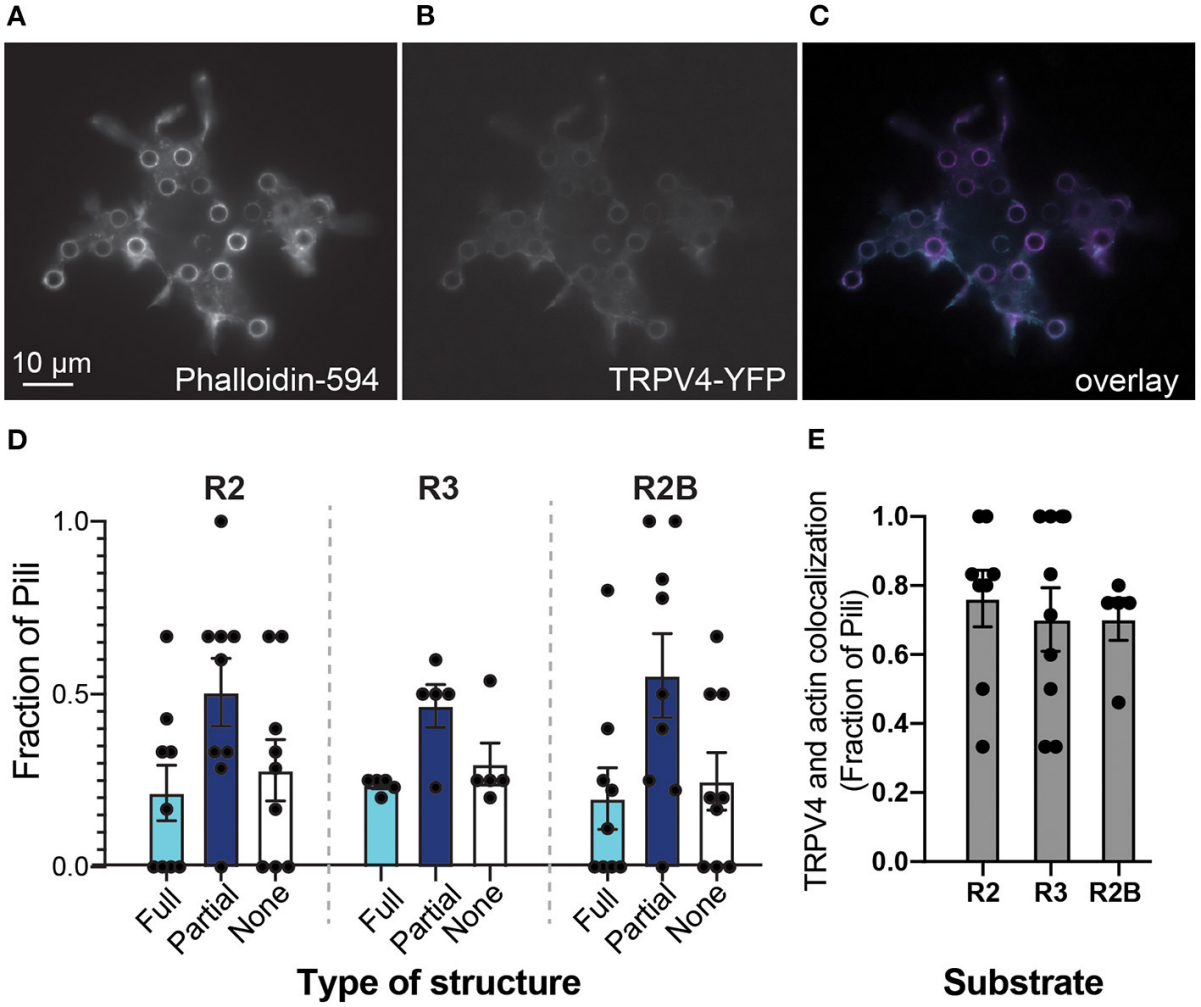
Substrate mechanics do not influence TRPV4 distribution. Epifluorescent images of HEK-293T cells expressing TRPV4 on R2 pillar array **(A)** filamentous actin structures were labeled with DyLight594-phalloidin **(B)** TRPV4-YFP signal **(C)** overlay of two channels, phalloidin signal in magenta and TRPV4-YFP in cyan. Note that both actin and TRPV4 condense at pillar-cell contacts, around the circumference of a subset of pili. **(D)** Structures at the edges of pili were scored as being full rings (Full), partial rings (Partial) or having no structure. There were no differences between arrays with distinct mechanics (two-way ANOVA). **(E)** As an indicator of colocalization of actin and TRPV4 at cell-pillar contacts, the number of pili subjacent to a cell exhibiting both TRPV4-YFP and actin structures were scored as a colocalization. There were no differences in these values when cells were cultured on substrates with distinct mechanics (Kruskal-Wallis test). Data are presented in mean ± s.e.m, with dots representing individual cells.

We first visualized TRPV4 distribution on pillar arrays, compared to actin-based structures. HEK-293T cells were transiently transfected with a plasmid encoding a TRPV4-YFP fusion protein and then actin structures were labeled using a post-fixation phalloidin stain. Fluorescent imaging from the cells showed that both TRPV4-YFP and actin form structures around individual pili (Figures 2A–C). Such a condensation of the TRPV4 channel around the edge of some pili subjacent to the cell is similar to the distribution of PIEZO1 and the PIEZO1-modulating protein STOML3 (Poole et al., 2014; Wetzel et al., 2017) on pillar arrays. For each cell, each pilus was scored according to whether the TRPV4 formed a ring around the total circumference of the pilus, a partial circumference or if there was no increase in signal around the pilus. The distribution of TRPV4 did not vary with the different substrates (Figure 2D) (two-way ANOVA), in contrast to our previously published observations for PIEZO1 (Bavi et al., 2019). As an indicator of colocalization of actin and TRPV4 at cell-pillar contacts, the number of pili subjacent to a cell exhibiting both TRPV4-YFP and actin structures were scored as a colocalization. This value was found to be variable from cell-to-cell, but not modulated by the mechanics and patterning of the subjacent array (Figure 2E).

To investigate whether the actin cytoskeleton impacts TRPV4-mediated currents, HEK-293T cells expressing TRPV4 were cultured on R3 pillar arrays and treated with cytochalasin D (2.5 μM), to disrupt actin filaments. MA currents in cytochalasin D treated cells were comparable with the control cells in response to pillar deflections ranging from 1 to 375 nm (Figure 1E). However, inhibiting actin polymerization reduced the current amplitude in response to larger stimuli, similar to the results acquired by manipulating the substrate roughness. We then tested whether depolymerization of microtubules modulates the MA currents. The stimulus-response plot was regenerated from the cells pretreated with 10 μM nocodazole. These data demonstrate that disruption of microtubules has no effect on TRPV4-mediated MA currents (Figure 1F). The current kinetics were unchanged in either cytochalasin D or nocodazole treated cells compared to the control (Supplementary Table 1).

### S824 Phosphorylation Modulates Mechanically Activation of TRPV4

**Figure 3:**
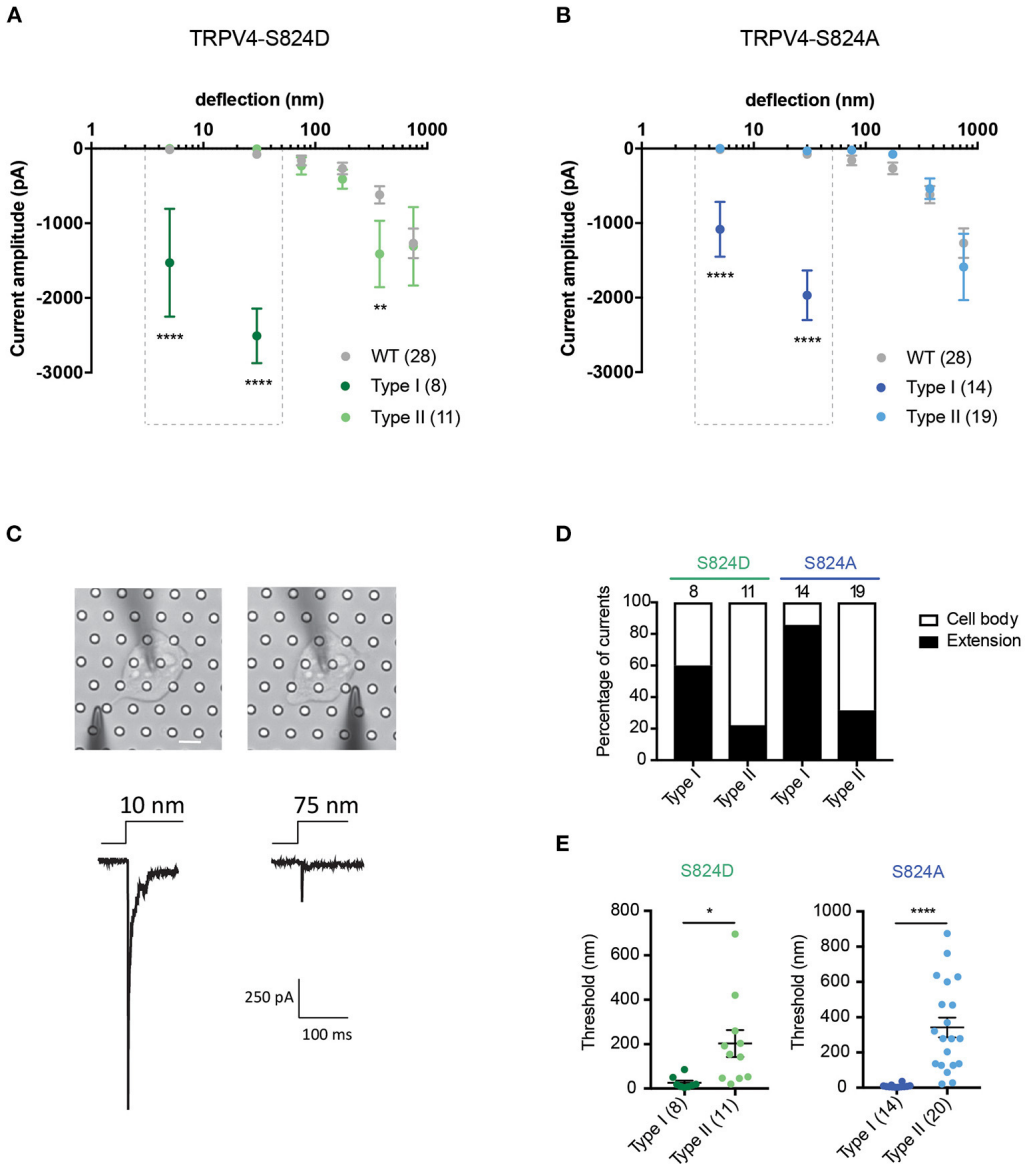
Phosphorylation site, S824 regulates TRPV4-mediated currents in response to pillar deflection. Stimulus-response plots for **(A)** TRPV4-S824D and **(B)** TRPV4-S824A in comparison to WT cultured on R3 substrate. Currents from TRPV4-S824D and TRPV4-S824A expressing cells were categorized into two types based on their sensitivity to deflection at a given pilus: Type I with high sensitivity and Type II with lower sensitivity. Ordinary two-way ANOVA with Sidak's multiple comparisons was used to analyze the plots. The first two bins (1–10 and 10–50 nm) were analyzed separately for Type I vs. Type II and Type I vs. WT as indicated in the dashed boxes (****p < 0.0001, Type I = 8 stimulation points vs. Type II = 11; WT = 28). Type II was compared to WT for all six bins (1–1,000 nm) and **p = 0.0083 for TRPV4-S824D for bin 250–500 nm (Type II = 11 stimulation points and WT = 28). **(C)** Bright-field image of a single HEK-293T cell expressing TRPV4-S824D and the corresponding current traces. Ten nanometer deflection of the pili subjacent to the cell extension resulted in current amplitude of 1,315 pA, while 75 nm deflection applied to the cell body of the same cell generated 265 nA current. **(D)** Percentage of type I and type II currents in TRPV4-S824D and TRPV4-S824A cells based on the site of deflection. The majority of Type I currents were observed upon deflection of the cell extensions rather than cell body. **(E)** Activation threshold for type I and type II currents in TRPV4-S824D and TRPV4-S824A cells was measured by averaging the smallest deflections generating currents. Data are presented as mean ± s.e.m, number in brackets indicates number of stimulation points (*p < 0.05). Full details on cell numbers outlined in Supplementary Table 1.

We next investigated whether mutations in the TRPV4 molecule itself impact substrate-deflection evoked currents. Previous studies have indicated the importance of the S824 residue in TRPV4 activation in response to various types of gating stimuli. Phosphorylation at S824 site enhances the activation of TRPV4 in response to arachidonic acid (AA), 4α-PDD and hypotonic swelling (Lee et al., 2010; Peng et al., 2010; Cao et al., 2018). To determine whether phosphorylation of TRPV4 affects channel mechanosensitivity, Flp-in T-REx HEK-293T cells containing a copy of the TRPV4 wild-type (WT) gene, or a gene encoding either the TRPV4 variant, TRPV4-S824D or TRPV4-S824A were analyzed. The TRPV4-S824D variant mimics the constitutive phosphorylation of the residue S824 whilst TRPV4-S824A is analogous to the non-phosphorylated state. Cells were cultured on R3 pillar arrays and stimuli were applied by deflecting pili subjacent to the cells. Mechanically evoked currents in cells expressing either TRPV4-S824D or TRPV4-S824A mutants exhibited two different types of sensitivities. The first population (classed as type I) were nano-ampere currents evoked in response to pillar deflections smaller than 50 nm and were therefore categorized as extremely sensitive. The second population of evoked currents (classed as type II) were comparable with those observed in cells expressing TRPV4-WT. As shown in Figure 3A, TRPV4-S824D-type I current amplitudes evoked from stimuli ranging between 1–10 and 10–50 nm were significantly larger compared to the amplitude of currents classed as TRPV4-S824D-type II or currents evoked in TRPV4-WT expressing cells (ordinary two-way ANOVA, Sidak's multiple comparison tests, *p* < 0.0001). These sensitive type I currents likely represent a large Ca^2+^ influx, which is known to result in rapid desensitization or cell toxicity. As such, stimulation with pillar deflections larger than 50 nm in cells where type I currents were evoked was not feasible due to cellular disruption. An activation threshold was calculated by noting the smallest stimulus that could evoke a current. The majority of type I currents were evoked by deflection of the pili subjacent to the extensions of the cell, while type II currents were mainly observed upon deflection of the pili under the cell body (Figures 3C,D). TRPV4-S824D-type I currents exhibited an activation threshold of 26.3 ± 9 nm compared with 203 ± 60 nm for TRPV4-S824D-type II currents (mean ± s.e.m). These data indicate that type I currents are more sensitive than type II (unpaired *t*-test, *p* = 0.02) (Figure 3E). Site-dependent sensitivity suggests a possible role of auxiliary molecules that interact with the phosphorylated channel, modulating its activity.

When we analyzed deflection-evoked currents in cells expressing TRPV4-S824A (unphosphorylated mimetic), we observed a similar phenomenon, i.e., two types of MA currents with distinct sensitivity (Figure 3B). The site of the deflection stimulus was also a determinant for these two distinct current types, as observed for the TRPV4-S824D variant. Namely, pillar deflections at peripheral regions of the cells expressing TRPV4-S824A resulted predominantly in type I currents, while type II currents were mostly evoked from regions near the cell body (Figure 3D). The average activation threshold for TRPV4-S824A-type I currents was 8.7 ± 2.3 nm, significantly lower than TRPV4-S824A-type II currents (341 ± 56 nm, mean ± s.e.m., unpaired *t*-test, *p* < 0.0001) (Figure 3E).

**Figure 4:**
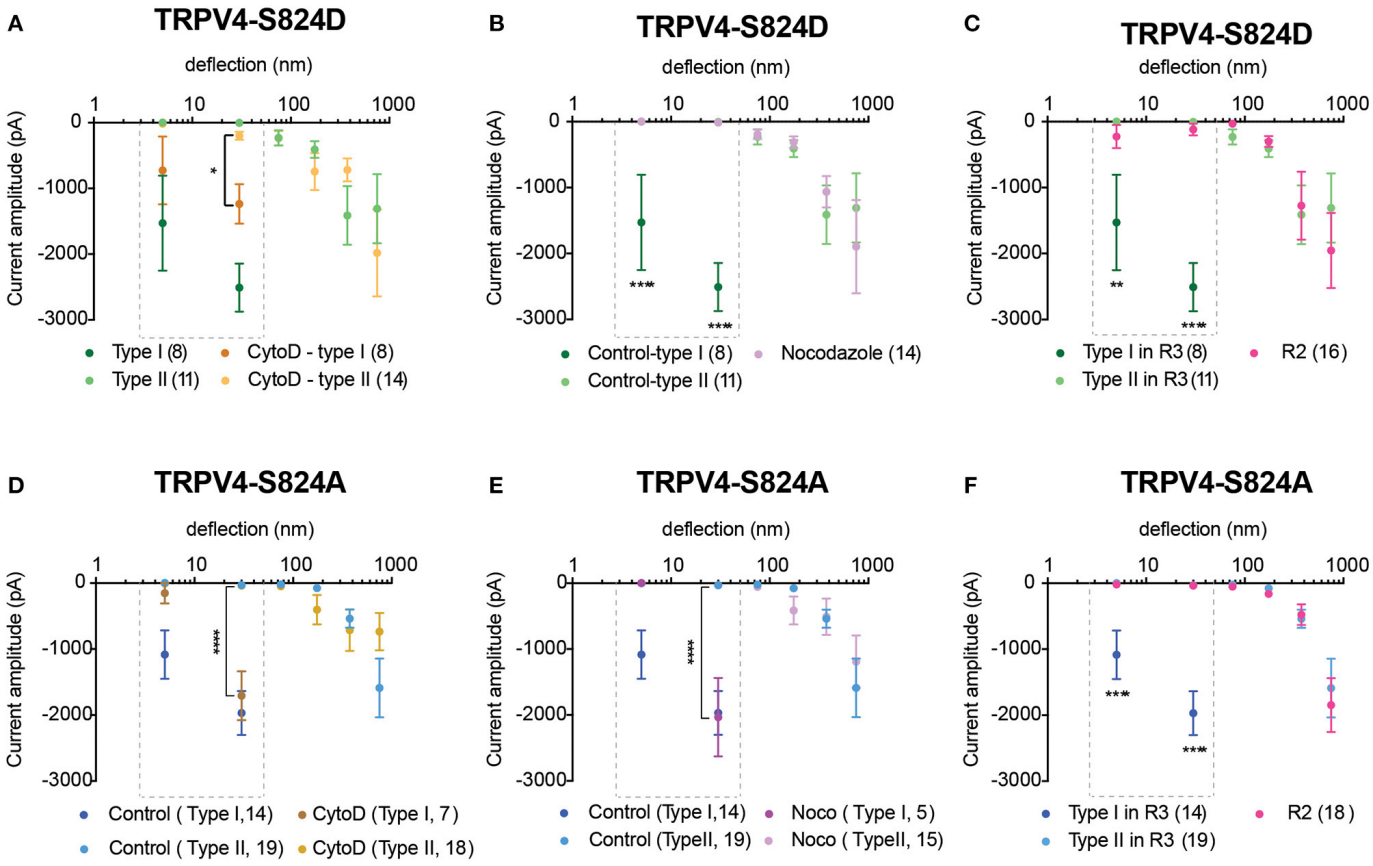
Impact of cytoskeleton and substrate mechanics on regulatory effect of S824 phosphorylation in TRPV4-mediated currents. Stimulus-response plots for TRPV4-S824D cells cultured on R3 substrate **(A)** after treatment with 2.5 μM cytochalasin D and **(B)** 10 μM nocodazole compared to the untreated control cells and **(C)** in comparison with TRPV4-S824D cells cultured on R2 substrate. Ordinary two-way ANOVA with Sidak's multiple comparisons was used to analyze the plots, *p = 0.018 in **(A)** (Type I control = 8 points, Type I cytochalasin = 11 points), ****p < 0.0001 in **(B)** (Type I control = 8, Nocodozole = 14 points), **p = 0.001 and ****p < 0.0001 in **(C)** (Type I, R3 = 8 points, R2 = 16). Stimulus-response plots for TRPV4-S824A cells cultured on R3 substrate **(D)** after treatment with 2.5 μM cytochalasin D and **(E)** 10 μM nocodazole compared to the untreated control cells and **(F)** in comparison with TRPV4-S824A cells cultured on R2 substrate. ****p < 0.0001 (R3, Type I = 14 stimulation points, R2 = 16). Data were analyzed using ordinary two-way ANOVA with Sidak's multiple comparisons. First two bins (1–10 and 10–50 nm) were analyzed separately for Type I currents as indicated in the dashed boxes. Data are presented in mean ± s.e.m., number in brackets indicates number of stimulation points. Full details on cell numbers outlined in Supplementary Table 1.

Previous studies suggest that phosphorylation of TRPV4 at residue S824 is required for its interaction with actin (Shin et al., 2015). As such, we next investigated whether actin cytoskeleton, microtubules or substrate stiffness are required in order to evoke type I currents. Cells expressing either TRPV4-S824D or TRPV4-S824A were cultured on R3 substrates in the presence of cytochalasin D or nocodazole in order to disrupt actin or microtubules, respectively. Compared to the control, cytochalasin D treated cells showed persistent sensitive MA currents in response to stimuli under 50 nm, indicating that type I currents are not dependent on TRPV4-actin interactions (Figures 4A,D). However, the depolymerization of microtubules with nocodazole abolished type I currents in cells expressing TRPV4-S824D, but not TRPV4-S824A (Figures 4B,E). Remarkably, the sensitive currents of both TRPV4-S824D and TRPV4-S824A expressing cells were absent when substrate roughness was increased by replacing R3 with R2 pillar arrays (Figures 4C,F). These data indicated that substrate roughness can modulate the sensitivity of S824D and S824A mechanically evoked currents, suggesting a regulatory role of TRPV4 phosphorylation in sensing substrate mechanics.

### MA Currents in TRPV4 Mutants Associated With Arthropathy

**Figure 5:**
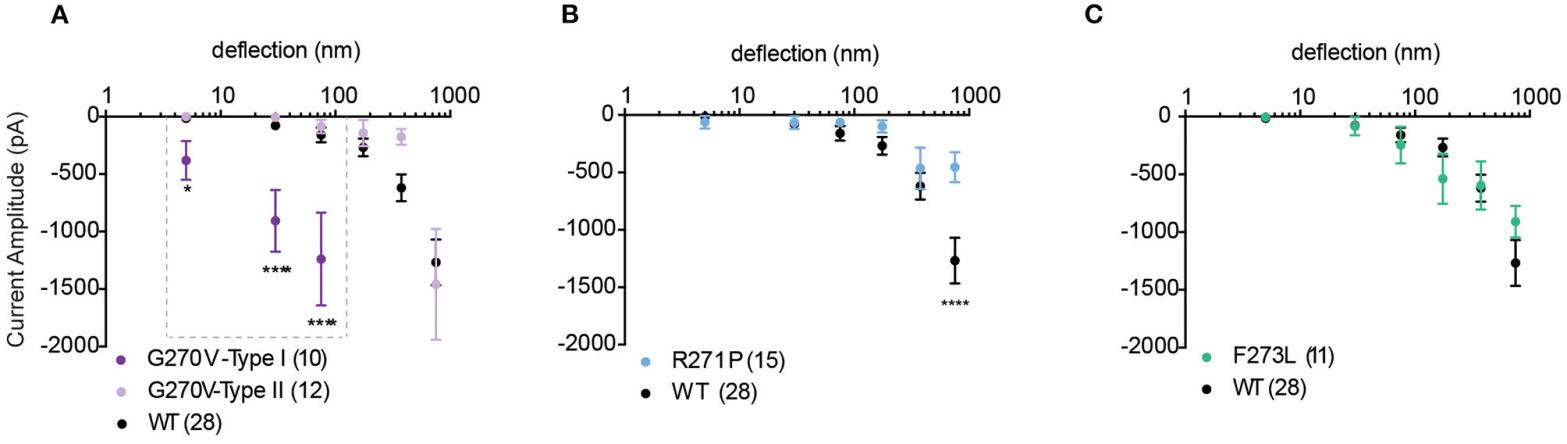
Mechanically activated currents in TRPV4 mutants associated with arthropathy in response to pillar deflection. Stimulus-response plots for **(A)** TRPV4-G270V, **(B)** TRPV4-R271P and **(C)** TRPV4-F273L in comparison to TRPV4-WT expressing cells cultured on R3 substrate. Data were analyzed using ordinary two-way ANOVA with Sidak's multiple comparisons. The first three bins in TRPV4-G270V curve (1–10, 10–50, and 50–100 nm) were analyzed separately for Type I vs. Type II as indicated in the dashed box. *p = 0.045 and ****p < 0.0001 in **(A)** (TRPV4-G270V, Type I = 10, Type II = 12 stimulation points). Data are presented as mean ± s.e.m., number in brackets indicates number of stimulation points. Full cell numbers outlined in Supplementary Table 1.

We next investigated whether mutations in TRPV4 previously described as loss-of-function mutations in response to non-mechanical stimuli also modulate substrate-deflection evoked TRPV4-mediated currents. Mutations in TRPV4 that are found within the ankyrin-repeat domain (TRPV4-G270V, -R271P, -F273L) have previously been shown to lead to inherited arthropathy in patients, and result in reduced Ca^2+^ influx in response to GSK1016790A and 4α-PDD in *in vitro* analyses of channel function (Lamandé et al., 2011). To characterize whether a similar loss-of-function was observed in response to substrate defection, currents evoked in cells expressing TRPV4-G270V, -R271P, -F273L cultured on R3 pillar arrays were analyzed. We found that, unlike activation with agonist, substrate deflection leads to robust channel gating in cells expressing TRPV4-G270V. Of the currents produced by stimuli within the range of 1–100 nm, 48% were sensitive type I currents in TRPV4-G270V expressing cells (11/23). These type I currents exhibited a significantly larger amplitude, compared with TRPV4-WT (Figure 5A). However, the F273L mutation had no effect on TRPV4-mediated currents evoked by pillar deflections (Figure 5C). Similarly, the R271P mutation did not significantly impact TRPV4-mediated currents in response to substrate deflections ranging from 1 to 500 nm. However, there was a reduction in current amplitude in cells expressing TRPV4-R271P when currents were evoked with stimuli ranging between 500 and 1,000 nm, compared to cells expressing TRPV4-WT (ordinary two-way ANOVA, Sidak's multiple comparison tests, *p* < 0.0001) (Figure 5B). These data stand in contrast to previous observations that demonstrate that these mutations cause a loss-of-function of agonist-evoked TRPV4-mediated currents. These findings support the notion that TRPV4 can be activated by distinct gating pathways depending on the types of input stimuli. It should be noted that, increasing the substrate roughness had no effect on the TRPV4-R271P, -F273L mediated currents but abolished the sensitive type I currents in TRPV4-G270V cells (Supplementary Figure 1).

We then used fluorimetric Ca^2+^ imaging to confirm the loss-of-function phenotype in TRPV4 channel activity in response to GSK1016790A. The Ca^2+^ response was significantly reduced in TRPV4-G270V, -R271P, -F273L mutants, compared to TRPV4-WT, consistent with previously published data (Lamandé et al., 2011). The Ca^2+^ response in cells expressing TRPV4-S824A and -S824D variants were comparable to TRPV4-WT controls (Supplementary Figure 2) and as previously reported (Cao et al., 2018). We then confirmed the impact of these mutations on channel localization at the plasma membrane, using cell-surface biotinylation (Supplementary Figure 2). These data indicate decreased membrane localization of TRPV4-G270V, -R271P, -F273L mutants compared to TRPV4-WT. Reduced levels of channel on the surface of the cell can account for diminished channel activity in response to TRPV4 agonists. However, substrate-deflection analysis shows that stimulation of these mutants produces currents of comparable magnitude to TRPV4-WT. These data suggest that the channels themselves are not loss-of-function mutants in response to mechanical stimulation when compared with TRPV4-WT.

## Discussion

We have previously shown that TRPV4 can be directly activated by mechanical stimuli applied via the substrate, mimicking tensile forces applied to cells via connections with their microenvironment. Here, we addressed the impact of the mechanical properties of the underlying substrate and intracellular cytoskeletal structures on mechanical activation of TRPV4 at the cell-substrate interface. Our data support our previous findings that TRPV4 has a distinct mechanotransduction profile when compared to the PIEZO1 channel. PIEZO1-mediated currents are efficiently evoked using high-speed pressure clamp, cellular indentation and pillar deflections, whereas TRPV4-mediated currents were only evoked by substrate deflections, but not indentation nor membrane stretch applied with HSPC (Servin-Vences et al., 2017). In previous work we have modeled the changes in membrane tension that are induced by these different stimulation techniques and while there is a moderate change in membrane tension from pillar deflection, the predicted values are significantly lower than those predicted to arise from the use of HSPC or cellular indentation (Bavi et al., 2019). Here we demonstrate that manipulating the mechanical properties of the substrate does not modulate TRPV4-mediated currents in response to substrate deflections. In addition, disruption of cytoskeletal elements including actin and microtubules had minimal effect on TRPV4 activation in this assay. In contrast, deflection-evoked PIEZO1 currents were previously shown to be modulated by the mechanics of the substrate, in an actin-dependent manner (Bavi et al., 2019). Similarly, activation of PIEZO1 by cell-generated forces is dependent on substrate stiffness and actomyosin traction forces (Pathak et al., 2014; Ellefsen et al., 2019). Thus, not only do these two channels exhibit distinct activation profiles in response to different mechanical inputs, but the currents evoked by substrate deflections are modulated by different mechanical and molecular factors. The data presented here demonstrate that, in a similar cellular background, substrate mechanics differentially impact TRPV4 vs. PIEZO1 activation. However, given the fact that our data indicates that the context of channel activation can be a critical modulatory factor, we cannot definitively conclude that these observations would hold true across all cells and tissues.

The mechanical activation of TRPV4 is not only distinct from the activation of PIEZO1, but data presented here on the impact of point mutations within the TRPV4 sequence suggest that mechanical activation of TRPV4 is distinct from activation of the channel by other gating stimuli. Channel activation of TRPV4-S824D and TRPV4-S824A compared to TRPV4-WT is profoundly dependent on the type of gating stimulus that is applied. TRPV4-dependent Ca^2+^ signaling, measured using fluorimetric Ca^2+^ dyes, is increased in cells expressing TRPV4-S824D and decreased in cells expressing TRPV4-S824A in response to AA, in comparison with the WT channel. In contrast, both mutations lead to sensitization of TRPV4 in response to 4α-PDD and heat (Lee et al., 2010) and neither mutation impacts TRPV4 channel activation by the TRPV4 agonist GSK1016790A (Cao et al., 2018). However, when we assayed the impact of mutations in TRPV4 on deflection-evoked currents, two distinct types of currents were generated when the S824 residue in TRPV4 was manipulated to either mimic constitutive phosphorylation (S824D) or prevent phosphorylation (S824A). Type I currents were extremely sensitive (with an amplitude over 1,000 pA) and were evoked by molecular-scale deflections (below 50 nm). Type II currents gradually scaled in response to 1–1,000 nm stimuli with a comparable sensitivity to TRPV4-WT. The average pillar deflection required for TRPV4 activation was 196 nm, comparable with the stimulus size for channel gating in chondrocytes and nociceptors with high mechanical threshold (Poole et al., 2014; Servin-Vences et al., 2017). However, the deflection threshold was decreased by one order of magnitude in type I currents of TRPV4-S824D and TRPV4-S824A. The sensitivity of these currents was similar to the currents from DRG mechanoreceptors with low threshold required for fine touch (Poole et al., 2014).

Changes in substrate mechanics and the cytoskeleton did not modulate deflection-evoked TRPV4-WT currents. However, increasing substrate roughness inhibited type I currents in cells expressing either TRPV4-S824A or TRPV4-S824D. These data suggest that the phosphorylation of TRPV4 at S824 can influence the impact of changing substrate mechanics on channel activation. Previous studies revealed that TRPV4 plays a central role in mechanotransduction during dynamic loading. Pharmacological inhibition of TRPV4 prevents mechanically induced extracellular matrix biosynthesis, while stimulation of TRPV4 enhanced matrix production in the absence of mechanical loading (O'Conor et al., 2014). As the pillar array assay most closely mimics forces applied to cells via surrounding matrices *in vivo*, our data suggest that matrix mechanics may also regulate TRPV4 activation but such regulation requires specific conditions such as phosphorylation at S824, or mutation to a non-phosphorylatable state. An open question that remains to be addressed is whether other modifications to the TRPV4 sequence or in associated molecules can also result in similar effects.

It has been previously proposed that the C-terminal domain of TRPV4 forms an autoinhibitory complex with a positively-charged domain in the amino tail (Strotmann et al., 2010) that is dissociated by calmodulin (CaM) binding. Mutation of E797-D798 showed an increase in basal activity of TRPV4. Unlike wild-type that is robustly activated by CaM, this mutant is not responsive to Ca^2+^-CaM binding. It has been suggested that the E797 and D798 residues are required for a salt bridge that maintains the closed state of the channel, while CaM binding to the adjacent site interferes with this inhibition (Loukin et al., 2015). Furthermore, stromal interaction molecule 1 (STIM1), a Ca^2+^ influx regulator, can interact with the C-terminal tail of TRPV4. It has been reported that STIM1 association inhibits TRPV4 activity. However, phosphorylation of S824 residue prevents the interaction between TRPV4 and STIM1, resulting in TRPV4 gain-of-function, in response to application of 4aPDD (Shin et al., 2015). Phosphorylation of TRPV4 residue S824 has also been shown to lead to increased Ca^2+^ signaling via the TRPV4 channel in response to hypertonic stimuli (Fan et al., 2009). While the cell swelling that occurs in response to a hypotonic solution is a mechanical stimulus at the cellular level, the resulting activation of TRPV4 relies on a second messenger (via PLA2). We have previously shown that TRPV4 activation via pillar arrays is a distinct process whereby the second messenger is not required (Servin-Vences et al., 2017). The increase activity of S824D and S824A in response to mechanical stimuli may be accounted for by similar disinhibition mechanisms through association or dissociation of auxiliary proteins or destabilizing the channel closure by disrupting a bond. However, additional research is required to investigate this further, particularly given the importance of context and gaiting stimulus in the activation of this polymodal channel.

The arthropathy-associated mutants including TRPV4-G270V, -R271P and -F273L exhibit loss-of-function phenotype in response to application of TRPV4-specific agonist (confirmed here) and failed to respond to hypotonic cell swelling (Lamandé et al., 2011). However, when stimuli were applied via substrate deflection, these mutant channels sustained currents comparable to TRPV4-WT. In the case of TRPV4-G270V, an increase in sensitivity was observed. Using cell surface biotinylation, we confirmed impaired membrane localization of these mutants. Hence, the reduced calcium influx that was observed with agonist or cell swelling might be associated with the low membrane expression level, resulting in reduced current density. However, with the pillar array assay, stimuli are applied to a delimited region of the membrane, where channels are arrayed at the edge of individual pili. Our data suggests that the actual mechanical activation of TRPV4 is not inhibited by these point mutations. We have previously demonstrated that TRPV4 activation by substrate deflections does not depend on PLA2 (Servin-Vences et al., 2017), in contrast to activation of TRPV4 by hypotonic cell swelling (a distinct type of mechanical stimulus). The differential impact of these point mutations on TRPV4 activity (loss-of-function in response to hypotonic cell swelling vs. no change or gain-of-function in response to substrate deflections) supports the hypothesis that activation of TRPV4 by these two distinct types of mechanical stimuli are separable processes.

Previous studies have suggested distinct roles for TRPV4 mechanotransduction in osteoarthritis (OA) subtypes. Cartilage-specific deletion of TRPV4 prevents age-related OA in adulthood but had no effects on injury-associated OA (O'Conor et al., 2016). In addition, loss of TRPV4 in pan-knockout mice accelerates age-related OA development (Clark et al., 2010). These findings imply that TRPV4 plays a critical role in mechanical signaling in a temporal and tissue-specific manner. Therefore, in diseases such as OA and arthropathy, epigenetic changes during aging may modulate mechanotransduction, and thus the regulatory effects of arthropathy mutants on downstream pathways may occur over a longer term, as the mechanics of the cellular microenvironment change with disease progression. The synergistic effects of point mutations within TRPV4 and both cell intrinsic (microtubules) and cell extrinsic (substrate mechanics) factors highlight the importance of considering context when studying mechanical activation of ion channels. This study addressed two important elements of the cell-substrate interface, the mechanics of the substrate and cytoskeletal structures within the cell. Future studies will be required to determine whether additional molecules that are present in this compartment are involved in mechanical signaling via TRPV4, including extracellular matrix molecules, cell binding and mechanosignalling molecules such as the integrins and additional intracellular force-sensing proteins such as those found in focal adhesion structures.

Together, our findings suggest that mechanical activation of TRPV4 exhibits distinct modulatory mechanisms, compared to PIEZO1. Ion channels with diverse activation and modulation profiles may enable cells to differentiate and probe their complex and changing mechanical microenvironment and respond accordingly. In addition, our data highlight the fact that activation of TRPV4 is extremely dependent on the gating stimulus. It is yet to be determined whether the pathways downstream of the channel activation can be also regulated by divergent gating stimuli.

## Data Availability Statement

The original contributions presented in the study are included in the article/Supplementary Material, further inquiries can be directed to the corresponding author/s.

## Author Contributions

SS collected electrophysiology and calcium imaging data. LS conducted cell surface biotinylation. JR and KP collected imaging data. AT established electrophysiology conditions. SL provided essential tools. Manuscript was written by SS and KP with input from all authors. All authors contributed to the article and approved the submitted version.

## Conflict of Interest

The authors declare that the research was conducted in the absence of any commercial or financial relationships that could be construed as a potential conflict of interest.
