# Supplementary material for: Modulating the Mechanical Activation of TRPV4 at the Cell-Substrate Interface
Source: Front Bioeng Biotechnol. 2021 Jan 18;8:608951. doi: 10.3389/fbioe.2020.608951 (PMC7848117; doi:10.3389/fbioe.2020.608951)
Supplement: Supplementary file 1 [file Table_1.DOCX]

|  | Property | R3 | R2 | Cytochalasin D | Nocodazole |
| --- | --- | --- | --- | --- | --- |
| WT | Latency (ms)  τ_1_ (ms)  τ_2_ (ms) | 1.3 ± 0.02  0.3 ± 0.02  7.9 ± 1.4  (28 cells) | 1.2 ± 0.02  0.3 ± 0.02  8.2 ± 2.9  (13 cells) | 1.2 ± 0.02  0.2 ± 0.03  5.7 ± 2.8  (7 cells) | 1.2 ± 0.02  0.3 ± 0.03  11 ± 3.1  (11 cells) |
| S824D | Latency (ms)  τ_1_ (ms)  τ_2_ (ms) | 1.3 ± 0.02  0.2 ± 0.02  8.9 ± 4.7  (15 cells) | 1.2 ± 0.02  0.2 ± 0.01  7.7 ± 1.2  (16 cells) | 1.3 ± 0.05  0.3 ± 0.03  7.6 ± 1.8  (16 cells) | 1.2 ± 0.02  0.2 ± 0.03  6.2 ± 2.6  (14 cells) |
| S824A | Latency (ms)  τ_1_ (ms)  τ_2_ (ms) | 1.2 ± 0.02  0.2 ± 0.02  3.9 ± 1.3  (20 cells) | 1.1 ± 0.01  0.3 ± 0.01  9.9 ± 3.9  (18 cells) | 1.3 ± 0.05  0.3 ± 0.07  5.3 ± 1.3  (17 cells) | 1.2 ± 0.3  0.3 ± 0.04  7.6 ± 2.1  (12 cells) |
| G270V | Latency (ms)  τ_1_ (ms)  τ_2_ (ms) | 1.3 ± 0.05  0.3 ± 0.03  6.2 ± 2.7  (14 cells) | 1.4 ± 0.01  0.3 ± 0.09  8.6 ± 3.2  (11 cells) |  |  |
| R271P | Latency (ms)  τ_1_ (ms)  τ_2_ (ms) | 1.2 ± 0.02  0.2 ± 0.02  8.8 ± 2.1  (15 cells) | 1.2 ± 0.03  0.2 ± 0.04  6.3 ± 4.9  (9 cells) |  |  |
| F273L | Latency (ms)  τ_1_ (ms)  τ_2_ (ms) | 1.4 ± 0.01  0.2 ± 0.02  9.4 ± 2.6  (11 cells) | 1.2 ± 0.02  0.3 ± 0.05  3.4 ± 1.4  (12 cells) |  |  |

|  | Property | R2 - stiff | R2B |
| --- | --- | --- | --- |
| WT | Latency (ms)  τ_1_ (ms)  τ_2_ (ms) | 1.1 ± 0.01  0.2 ± 0.06  6.4 ± 3.1  (9 cells) | 1.2 ± 0.02  0.4 ± 0.08  8.1 ± 3.2  (11 cells) |

**Table S1.** Physiological properties of the mechanically activated currents in HEK-293T cells expressing WT or the mutant TRPV4 variants. Cells were cultured on R3, R2 or R2B substrates. Cells treated with cytochalasin D or nocodazole were cultured on R3 substrate. The latency was quantified as the time delay between the stimulus application and onset of the current. The activation time constant (τ_1_) and inactivation time constant (τ_2_) were calculated by a mono-exponential fit of the current rise and current decay respectively. Data are presented as mean ± s.e.m.
