# Supplementary material for: Modulating the Mechanical Activation of TRPV4 at the Cell-Substrate Interface
Source: Front Bioeng Biotechnol. 2021 Jan 18;8:608951. doi: 10.3389/fbioe.2020.608951 (PMC7848117; doi:10.3389/fbioe.2020.608951)
Supplement: Supplementary file 2 [file Image_1.PDF]

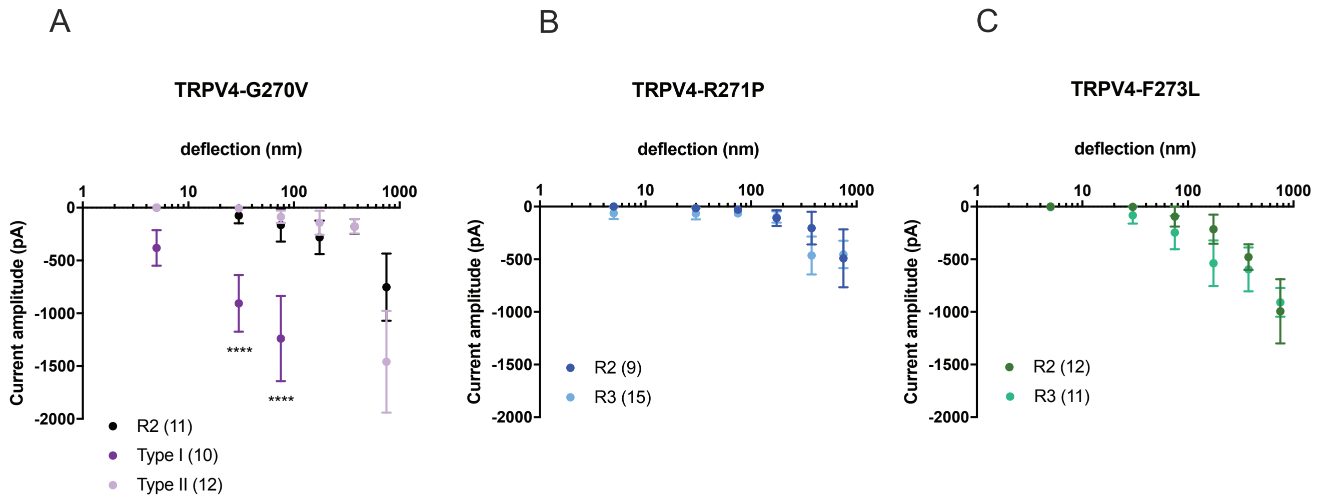

Figure S1. Mechanically activated currents in TRPV4 mutants associated with arthropathy in response to pillar deflection. Stimulus-response plots for (A) G270V, (B) R271P and (C) F273L cultured on R3 substrate compared to the cells cultured on R2. ****p<0.0001 in A. Data were analysed using ordinary two-way ANOVA with Sidak's multiple comparisons. First three bins in G270 (1-10 nm, 10-50 nm and 50-100 nm) were analysed separately for Type I and Type II in R3 vs R2 as indicated in the dashed box. Data are presented in mean ± s.e.m.
