# Supplementary material for: Modulating the Mechanical Activation of TRPV4 at the Cell-Substrate Interface
Source: Front Bioeng Biotechnol. 2021 Jan 18;8:608951. doi: 10.3389/fbioe.2020.608951 (PMC7848117; doi:10.3389/fbioe.2020.608951)
Supplement: Supplementary file 3 [file Image_2.PDF]

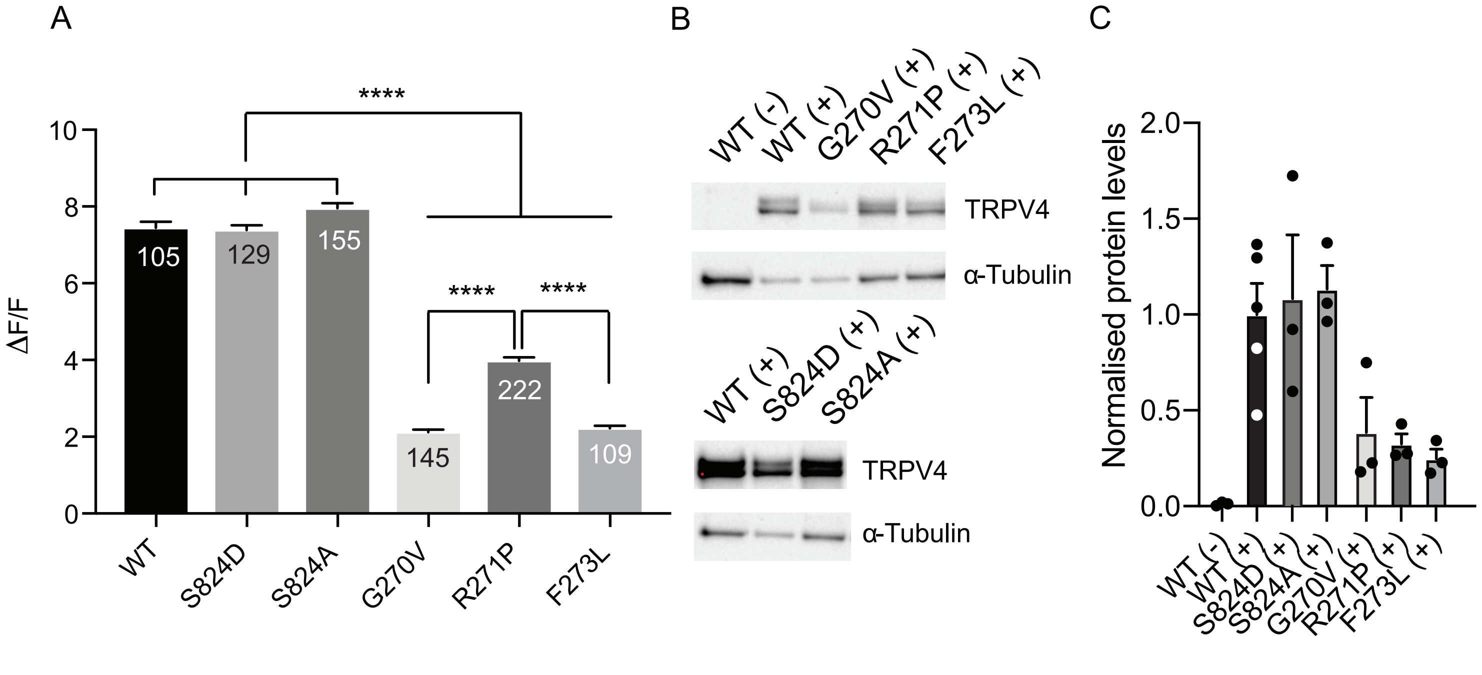

Figure S2: Fluorimetric Ca^{2+} imaging of HEK-293T cells expressing TRPV4 variants. (A) HEK-293T cells expressing TRPV4- WT, -S824D, -S824A, -G270V, -R271P, and -F273L were perfused with 1 μM of GSK1016790A and peak $[Ca^{2+}]$ was monitored using Cal520 Ca^{2+} responsive dye. A comparison of ΔF/F0 indicated significantly lower Ca^{2+} in cells expressing TRPV4-G270V, -R271P, and -F273L in comparison with TRPV4-WT. Data were analyzed using one-way ANOVA with Tukey's multiple comparison,****$p<0.0001$. Data are presented as mean ± s.e.m. and represent data collected from 2 separate experiments. (B) Western blot of TRPV4 protein found in the plasma membrane fraction, isolated as a biotinylated fraction. (C) Corresponding quantification of TRPV4 levels from Western blot analysis. Data are presented as mean ± s.e.m with individual data points highlighted as dots (n = 3 separate experiments).
